# Antiphospholipid antibody formation during immune checkpoint inhibition in patients with cancer: a prospective cohort study

**DOI:** 10.1016/j.rpth.2026.106654

**Published:** 2026-05-19

**Authors:** Anniek Strijdhorst, Dorien M. Salet, Jente M. Schoenaker, Vossa van der Vegte, Tom T.P. Seijkens, Hanneke W.M. van Laarhoven, Rolf T. Urbanus, Nick van Es

**Affiliations:** 1 Department of Vascular Medicine, Amsterdam University Medical Center, location University of Amsterdam, Amsterdam, The Netherlands; 2 Cancer Center Amsterdam, Cancer Treatment and Quality of Life, Amsterdam, The Netherlands; 3 Amsterdam Cardiovascular Sciences, Atherosclerosis & Ischemic Syndromes, Amsterdam, The Netherlands; 4 Center for Benign Haematology, Thrombosis and Haemostasis, Van Creveldkliniek, University Medical Center Utrecht, Utrecht University, Utrecht, The Netherlands; 5 Division of Medical Oncology, Netherlands Cancer Institute, Amsterdam, The Netherlands; 6 Department of Medical Oncology, Amsterdam University Medical Center, location University of Amsterdam, Amsterdam, The Netherlands

**Keywords:** antiphospholipid antibodies, arterial thromboembolism, immune checkpoint inhibitor, thrombin generation assay, venous thromboembolism

## Abstract

**Background:**

Immune checkpoint inhibitors (ICIs) significantly improve survival in many cancer types. However, ICIs are associated with an increased risk of venous and arterial thromboembolism, and the underlying mechanisms are incompletely understood. We hypothesized that ICI therapy may induce the development of antiphospholipid antibodies, contributing to hypercoagulability.

**Objectives:**

To evaluate changes in antiphospholipid antibody positivity and thrombin generation in patients with cancer initiating ICI therapy.

**Methods:**

Data were used from ITHACA, a prospective cohort study including adults with cancer who underwent blood sampling at baseline and 3 months after starting ICI therapy. At both time points, lupus anticoagulant, anti–β2-glycoprotein I immunoglobulin (Ig)G/IgM, anticardiolipin IgG/IgM, and thrombin generation were measured. The primary outcome was the development of antiphospholipid antibody positivity at 3 months. Secondary outcomes included changes in thrombin generation (endogenous thrombin potential and peak height) and thromboembolic events.

**Results:**

Forty patients initiating anti–programmed cell death protein 1 or anti–cytotoxic T-lymphocyte–associated antigen 4 therapy were included. The median age was 63 years (IQR, 57-69); 80% were male, and 5% were on anticoagulation. At baseline, 3 patients (8%) had positive anti–β2-glycoprotein I or anticardiolipin antibodies, compared with 2 patients (5%) at 3 months (*P* = 1.00). No patients had a positive lupus anticoagulant. Endogenous thrombin potential (935 vs 949 nM ∗ min; *P* = .65) and peak height (277 vs 279 nM; *P* = .49) were similar at both time points. One patient developed venous thromboembolism.

**Conclusion:**

ICI therapy was not associated with the development of antiphospholipid antibodies or changes in thrombin generation after 3 months. These findings suggest that the short-term risk of thromboembolic events after initiation of ICI therapy may not be mediated by antiphospholipid antibodies.

## Essentials

• ICIs are associated with (arterial) thromboembolic events.
• Blood samples from 40 patients were assessed before and after 3 months of ICI therapy.
• No development of antiphospholipid antibodies was observed after 3 months of ICI therapy.
• Thrombin generation did not change after 3 months of ICI therapy.

## 1 Introduction

Immune checkpoint inhibitors (ICIs) have revolutionized cancer treatment by enhancing T-cell activation and improving survival in various malignancies [1]. However, immune-related adverse events (irAEs) occur in up to 50% of patients [2]. Emerging evidence suggests that ICI treatment is associated with a 2- to 3-fold increase in the risk of venous thromboembolism (VTE) and arterial thromboembolism (ATE) compared with patients not receiving ICIs [[3], [4], [5]].

Although the mechanisms underlying this increased risk remain incompletely understood, activation of the coagulation cascade by systemic inflammation, often called immunothrombosis, may play a role [6]. However, while some experimental data suggest that ICIs can induce platelet activation *in vitro* and increase tissue factor (TF) expression in supernatants from T-cell and cancer cocultures, clinical evidence for ICI-induced hypercoagulability remains inconclusive [7]. Previous studies have not shown consistent changes in global coagulation markers, such as D-dimer and thrombin peak height, following ICI treatment [[7], [8], [9]]. Further investigation into the factors contributing to the risk of both VTE and ATE in ICI-treated patients is needed.

Autoimmunity is a hallmark of irAEs, as ICI therapy enhances T-cell activation and disrupts immune tolerance [2]. Even without overt irAEs, immune activation by ICIs may also promote the development of autoantibodies, such as antiphospholipid antibodies (aPLs). Previous studies reported the development of autoantibodies following ICI therapy, which have been associated with the occurrence of irAEs [10]. In addition, autoantibodies have been detected in approximately one-third of patients with irAE encephalitis, further suggesting a role for B-cell activation in irAE pathogenesis [11]. The presence of aPLs is associated with both VTE and ATE, as these antibodies can affect the endothelium and immune cells, leading to processes such as neutrophil extracellular trap formation and TF activation [12,13]. Mechanistic studies suggest that immune activation, T-cell proliferation, and increased TF expression may promote aPL generation [[14], [15], [16]]. While case series have described severe antiphospholipid syndrome-like syndromes after ICI initiation, aPLs may also form subclinically and subsequently contribute to the risk of thrombosis [17].

We hypothesized that ICI-induced aPL formation could contribute to increased thrombotic risk. Therefore, the objective of this study was to investigate the development of aPLs after ICI treatment and to assess their potential association with thrombin generation parameters.

## 2 Methods

### 2.1 Study design and study group

This was a predefined analysis of ITHACA, an ongoing, prospective, single-center, observational cohort study conducted at Amsterdam University Medical Center (clinicaltrials.gov: NCT06519292). The primary aim was to assess changes in atherosclerotic noncalcified plaque volume from baseline to 1 year using coronary computed tomography angiography. Patients with cancer (≥50 years) scheduled to start ICI therapy were eligible. Exclusion criteria included ICI use ≤12 months, chronic infection requiring antibiotics, autoimmune disease requiring immunosuppression, or contraindications to computed tomography (eg, atrial fibrillation, estimated glomerular filtration rate < 30 mL/min/1.73 m^2^, or contrast allergy). Blood was collected at baseline, 3 months, and 1 year. This study was approved by the local medical ethics committee, and all patients provided written informed consent.

For this analysis, the first 40 patients with evaluable blood samples at baseline and at 3 months were included. Data on demographics, cardiovascular risk factors, cancer characteristics, medications, and clinical outcomes (grade ≥ 2 irAEs [18], ATE, VTE, and all-cause mortality) were collected. Patients were followed until death, loss to follow-up, or July 1, 2025. Hypertension and dyslipidemia were defined as the use of blood pressure or lipid-lowering medication, respectively. This report adheres to the Strengthening the Reporting of Observational Studies in Epidemiology guidelines.

### 2.2 Plasma preparation

Blood was collected into citrate tubes, centrifuged at 1500 × *g* for 15 minutes, and the plasma was stored at −80 C. Before analysis, plasma was thawed at 37 C for 10 minutes, vortexed, and kept on ice.

### 2.3 aPLs

Plasma levels of anti–β2-glycoprotein I (anti-β2GPI) and anticardiolipin antibodies (ACAs; immunoglobulin [Ig]G and IgM isotypes) were measured with enzyme-linked immunosorbent assay QUANTA Lite Kits (INOVA Diagnostics) per the manufacturer’s instructions. ACA IgG/IgM levels >20 IgM phospholipid units (MPL; indeterminate: 12.5–20 MPL) and anti–β2GPI IgG/IgM levels >20 standard IgG anti-β2-glycoprotein I units (SGU) were considered positive. Values < 9 MPL/SGU were considered 9. Lupus anticoagulant (LAC) was screened using both HemosIL dRVVT Screen and Silica Clotting Time (Werfen), and confirmed on the MC10plus coagulometer (MERLIN Medical), mixed 1:1 with normal pooled plasma (NPP) and confirmed in accordance with current recommendations [19]. LAC was positive if the normalized ratio ([screen patient]/[screen NPP])/([confirm patient]/[confirm NPP]) was ≥1.2.

### 2.4 Thrombin generation

Thrombin generation was measured using calibrated automated thrombography in platelet-poor plasma at 20-second intervals for 1 hour, as previously described [20]. Coagulation was initiated with PPP-Reagent High (5 pM TF; Diagnostica Stago), with and without 40 nM rabbit thrombomodulin (TM; Prolytix), to assess activated protein C (APC) resistance [21]. APC sensitivity was calculated as the endogenous thrombin potential (ETP; or thrombin peak) with TM divided by ETP (or thrombin peak) without TM.

### 2.5 Study outcomes

The primary outcome was aPL positivity at 3 months. Secondary outcomes included changes in aPL titers, LAC ratio, ETP, thrombin peak, lag time, and the incidence of VTE (pulmonary embolism, deep venous thromboembolism, and other venous thromboses) and ATE (myocardial infarction, ischemic stroke, peripheral artery occlusion, and cardiovascular death).

### 2.6 Statistical analysis

Descriptive statistics summarized baseline characteristics. The change in the proportion of patients with aPL positivity between baseline and 3 months was compared using McNemar’s test. Changes in ETP, thrombin peak, aPL titers, and LAC were analyzed using paired *t*-tests or Wilcoxon signed-rank tests. To account for within-subject correlations and baseline values, a sensitivity analysis was performed using linear mixed-effects models with a random intercept. Subgroup analysis was performed by irAE status. Incidence rates were reported as proportions. Two-sided *P* values < .05 were considered statistically significant. No sample size calculation was performed due to a lack of prior data. Analyses were performed using R version 4.3.2 (R Foundation for Statistical Computing), and figures were created with GraphPad Prism version 10.0.0 (GraphPad Software).

## 3 Results and Discussion

Forty-four patients were enrolled between March 2023 and June 2024; of these, 40 had blood samples available at both baseline and 3 months and were included in the analysis. Four patients did not complete the 3-month sampling and were excluded. The median age was 63 years (IQR, 57-69), 80% of patients were male, 55% had stage II/III esophageal cancer, and 16 (40%) had distant metastases (Table). The median interval between sampling was 91 days (IQR, 84-104), during which patients received a median of 4 ICI cycles, either as adjuvant or palliative treatment, with or without concurrent chemotherapy. During a median follow-up of 16 months (IQR 12-29), no patients were lost to follow-up, and 9 (23%) died.

**Table:** Baseline characteristics.

Characteristics	ICI patients (*N* = 40) *n* (%)
Age (y), median (range)	63 (57-69)
Sex, male	32 (80)
Tumor type	
Esophageal cancer	22 (55)
Melanoma	16 (40)
Renal cell carcinoma	2 (5)
Metastasis at baseline	16 (40)
Type of ICI	
Anti–PD-1	36 (90)
Anti–PD-1 + anti–CTLA-4	4 (10)
History of VTE	2 (5)
History of ATE	8 (20)
Myocardial infarction	4 (10)
Coronary revascularization	2 (5)
Ischemic stroke	1 (3)
Transient ischemic attack	2 (5)
Medication use	
Direct oral anticoagulants	2 (5)
Platelet aggregation inhibitors	12 (30)
Immunosuppressive agents	3 (8)

ATE, arterial thromboembolism; CTLA-4, cytotoxic T-lymphocyte-associated protein 4; ICI, immune checkpoint inhibitor; PD-1, programmed cell death protein-1; VTE, venous thromboembolism.

**Figure 1:**
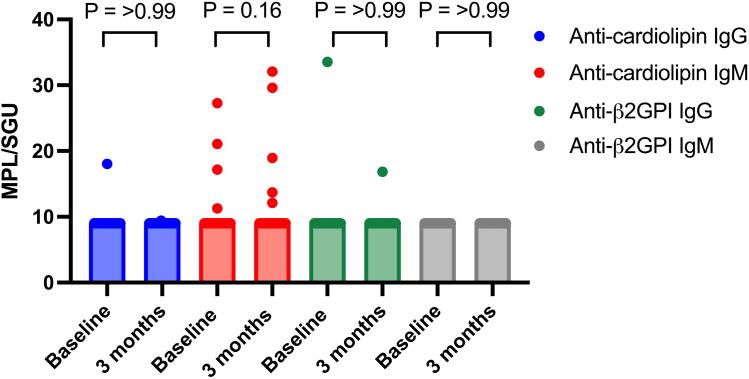
The difference in antiphospholipid antibody production between baseline and 3 months after immune checkpoint inhibitor initiation (*N* = 40). Bars represent the median, and dots represent individual patients. Anti-β2GP1, anti–β2-glycoprotein I; Ig, immunoglobulin; MPL, IgM phospholipid units; SGU, standard IgG anti-β2-glycoprotein I units.

The proportion of aPL-positive patients was similar at baseline (*n* = 3, 7.5%) and at 3 months (*n* = 2, 5%; *P* = 1.00). Thirty-five patients (88%) were negative at both time points, including 2 patients (5%) on anticoagulation. Three patients (7.5%) were aPL-positive at baseline (ACA IgG: 28 MPL; ACA IgM: 21 MPL; anti-β2GPI IgG: 34 SGU) but converted to aPL-negative or -indeterminate at follow-up. Of these, 1 had an active autoimmune disease, and 1 underwent recent major surgery. No patients had double- or triple-positive antibodies or LAC positivity. Two patients (5%) were aPL-positive at 3 months; of these, 1 converted from indeterminate ACA IgM (17 MPL) at baseline to positive ACA IgM (32 MPL), and 1 remained positive (27 MPL at baseline and 30 MPL at 3 months). When the cutoffs proposed in the new antiphospholipid syndrome (APS) classification criteria for research were used (intermediate-titer: 48-80; high-titer: >80), none of the patients were considered positive at baseline or at 3 months. No differences in continuous aPL titers between baseline and 3 months were observed (ACA IgM *P* = .16; all other aPLs *P* ≥ .99; Figure 1), and all LAC tests were negative (mean dilute Russell's viper venom time LAC ratio, 0.95 ± 0.04 vs 0.94 ± 0.05, *P* = .45; mean silica clotting time LAC ratio, 0.87 ± 0.07 vs 0.86 ± 0.09, *P* = .35). Sensitivity analysis using linear mixed-effects models confirmed these findings.

**Figure 2:**
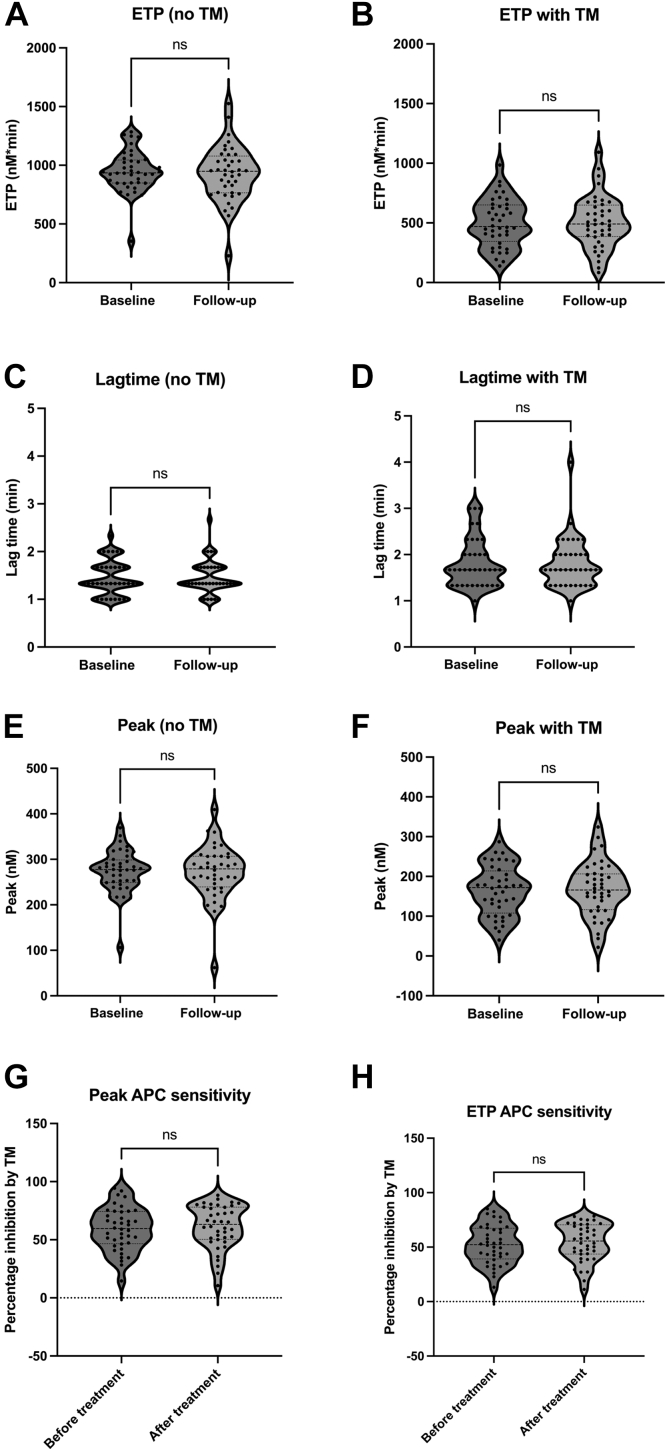
Thrombin generation parameters show no significant differences between baseline and 3 months after initiation of immune checkpoint inhibitors (*N* = 40). Endogenous thrombin potential (ETP) in (A) normal and (B) thrombomodulin (TM) conditions, assessing possible activated protein C (APC) resistance. Lag time (measured every 20 seconds) in (C) normal and (D) TM conditions. Peak thrombin generation in (E) normal and (F) TM conditions. APC sensitivity as a percentage inhibition of the (G) peak and (H) ETP. ns, not significant.

aPLs are associated with changes in thrombin generation and acquired APC resistance [22]. Thrombin generation was similar between baseline and follow-up, including no difference in median ETP (935 vs 949 nM ∗ min; *P* = .65), thrombin peak (277 vs 279 nM; *P* = .49), lag time (1.33 vs 1.33 min; *P* = .95), or APC sensitivity (median ETP APC: 52.2% vs 55.6%, *P* = .72; median peak APC: 59.6% vs 63.3%, *P* = .66; Figure 2). Findings were similar for patients with grade ≥ 2 irAEs (*n* = 16) and those without irAEs, both in normal and TM conditions (data not shown). Sensitivity analysis did not reveal significant differences in lag time, ETP, or peak between baseline and 3 months after accounting for baseline values.

One patient developed VTE (inferior caval vein thrombosis) after 11 months, with negative aPLs at baseline and indeterminate ACA IgM at 3 months (13.7 MPL). No other thromboembolic events were observed.

In this prospective cohort of 40 patients with cancer initiating ICIs, we did not find evidence of new aPL formation or increased thrombin generation after 3 months. No patients had double- or triple-positive antibodies or LAC positivity. The definition of aPL positivity remains debated, with recent American College of Rheumatology and European Alliance of Associations for Rheumatology guidelines recommending higher cutoffs (eg, 40-79 MPL for moderately positive and >80 MPL for highly positive) than those recommended by the manufacturer [19,23,24]. Given this, the low titers observed are unlikely to be clinically relevant or attributable to ICIs, and the proportion of aPL positivity was similar to that in the general population [25]. Furthermore, thrombin generation assay (TGA) parameters remained unchanged between baseline and 3 months.

Our data suggest that ICIs do not trigger aPL formation. Mechanistically, ICIs may promote aPLs formation via Th1-driven B-cell activation and the secretion of proinflammatory cytokines, such as interleukin-1β and tumor necrosis factor-α [14,16,26]. In murine models, loss of programmed cell death protein-1 signaling leads to lupus-like autoimmunity, suggesting that ICIs can unleash autoreactive T-cell responses [27]. Increased TF expression, observed in both cancer and ICI models *in vitro*, promotes aPL formation [28]. However, our clinical data do not support an effect of ICIs on aPL induction or coagulation activation within the first 3 months of therapy. Possible explanations include the need for a more specific or sustained immune trigger. For example, infections are known to induce transient aPL positivity, but this rarely leads to clinical APS [29,30]. Second, the development of APS-like syndromes following ICIs, as described in case reports, all occurring >6 months after ICI initiation, may represent rare irAEs rather than a common consequence [31]. Third, the interplay among tumor burden, host immune status, and ICI-induced immune activation is complex and may modulate the risk of aPL formation in ways that are not captured in our cohort.

Our TGA findings are consistent with previous studies on coagulation parameters. Vladic et al. [8] found no significant changes in P-selectin, D-dimer, or thrombin peak after ICI initiation (*n* = 15), although a trend was observed after 3 months. These findings indicate no evidence of increased hypercoagulability following ICI therapy.

Several limitations of the study should be acknowledged. Our modest sample size, limited number of longitudinal measurements, and relatively short follow-up period render our findings exploratory and may have limited statistical power to detect differences. In particular, the number of thrombotic events was too small to allow meaningful assessment of a potential association between aPL positivity and thrombotic outcomes. Nonetheless, the observation that none of the 40 patients had high antibody titers or LAC suggests that clinically relevant aPL formation is not frequent during ICI treatment. The 3-month observation period may have missed the later formation of aPLs, although autoimmunity typically occurs early after ICI initiation [2]. Most patients received ICIs as adjuvant treatment, which may not reflect the altered systemic inflammation and immunological milieu of advanced or metastatic disease, potentially affecting aPL risk [32]. Most patients received ICIs as adjuvant nivolumab for stage II to III disease, which may not reflect the broader population of patients receiving ICIs. As we enrolled only new ICI therapy users, our results may not be generalizable to patients who have received prior ICI therapy.

Our study provides prospective data on the short-term effects of ICI therapy on aPL formation and coagulation in patients with cancer. The absence of new aPL positivity and unchanged TGA parameters suggests that, in the majority of patients, ICI therapy does not induce a prothrombotic state via aPL formation. The increased risk of arterial events observed in other studies may be mediated by mechanisms such as atherosclerotic plaque destabilization and direct endothelial activation, whereas in VTE, endothelial and platelet activation, as well as other immune-mediated pathways, may play a more prominent role. Future studies should include larger, more diverse cohorts and longer follow-up with longitudinal measurements to assess potential long-term effects. Direct comparison of coagulation profiles, including TGA and aPL status, between patients with and without thrombotic complications in larger cohorts or case-control designs may help identify biomarkers and clarify pathophysiological pathways. Mechanistic investigations in animal models and *in vitro* could determine the conditions under which ICI therapy may induce aPL formation and alter the coagulation system.

In conclusion, while there is a mechanistic rationale for aPL induction, our data do not support a widespread effect of ICI therapy on aPL formation or thrombin generation in the short term. Further research is warranted to elucidate the pathways underlying ICI-associated thrombotic risk and to develop effective risk mitigation strategies for this patient population.
